# Supplementary figures and images for: Multinucleated giant cells are hallmarks of ovarian aging with unique immune and degradation-associated molecular signatures
Source: PLoS Biol. 2025 Jun 23;23(6):e3003204. doi: 10.1371/journal.pbio.3003204 (PMC12185119; doi:10.1371/journal.pbio.3003204)

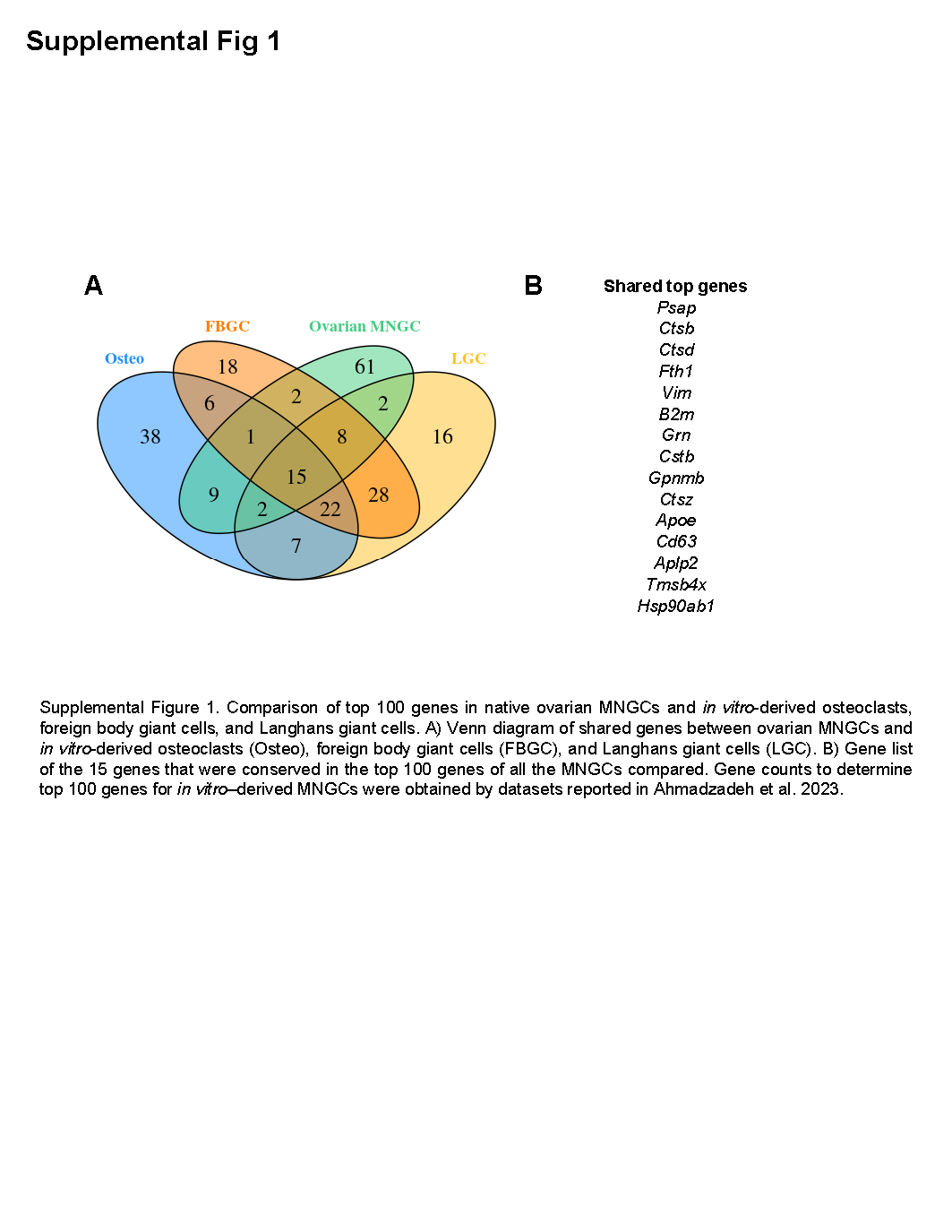

Supplement: S1 Fig — A) Venn diagram of shared genes between ovarian MNGCs and in vitro-derived osteoclasts (Osteo), foreign body giant cells (FBGC), and Langhans giant cells (LGC). B) Gene list of the 15 genes that were conserved in the top 100 genes of all the MNGCs compared. Gene counts to determine top 100 genes for in vitro–derived MNGCs were obtained by datasets reported in Ahmadzadeh and colleagues 2023 [24]. (TIFF) [file pbio.3003204.s001.tiff]

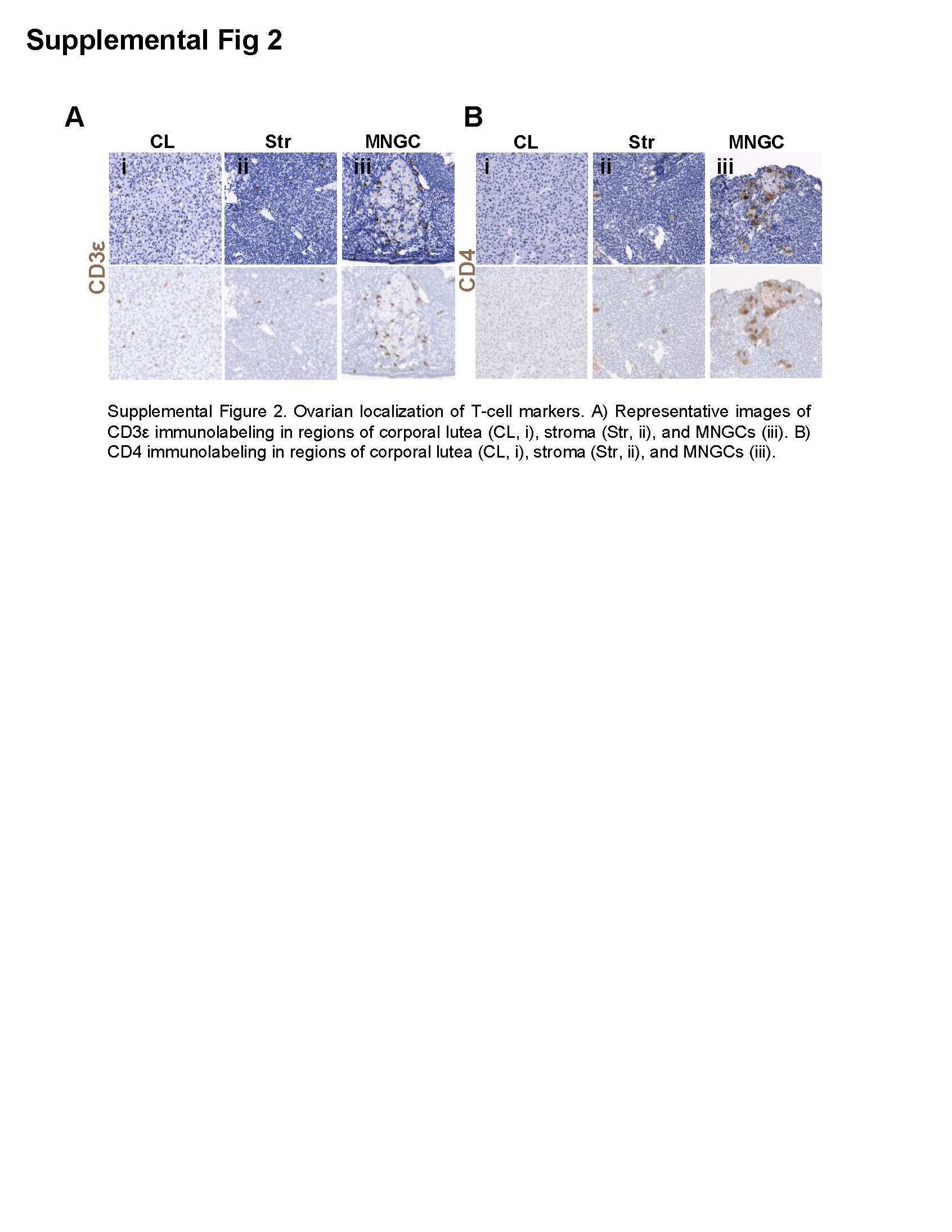

Supplement: S2 Fig — A) Representative images of CD3ε immunolabeling in regions of corporal lutea (CL, i), stroma (Str, ii), and MNGCs (iii). B) CD4 immunolabeling in regions of corporal lutea (CL, i), stroma (Str, ii), and MNGCs (iii). The data underlying the graphs can be accessed at https://doi.org/10.5061/dryad.kh18932j4. (TIF) [file pbio.3003204.s002.tif]

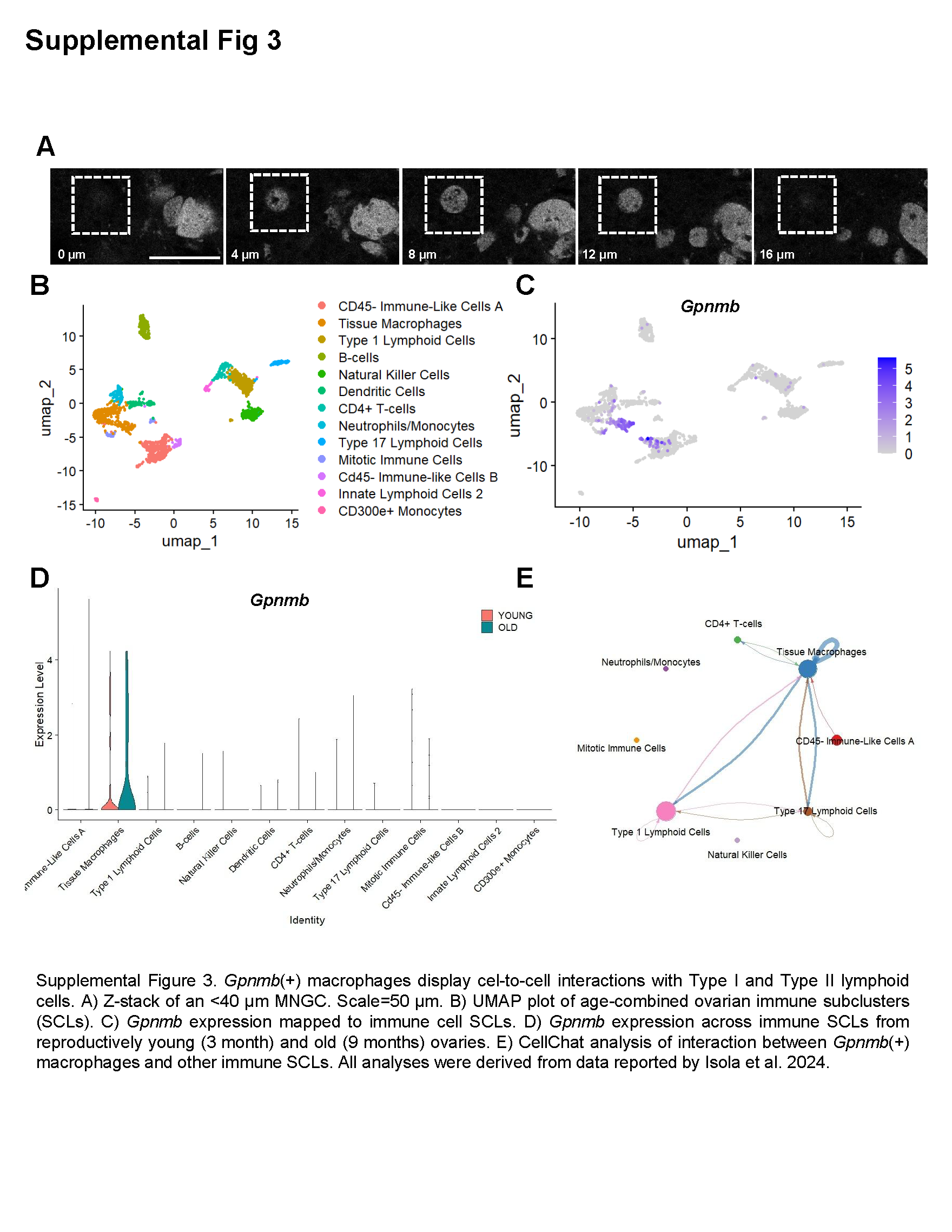

Supplement: S3 Fig — A) Z-stack of an < 40 µm MNGC. Scale = 50 µm. B) UMAP plot of age-combined ovarian immune subclusters (SCLs). C) Gpnmb expression mapped to immune cell SCLs. D) Gpnmb expression across immune SCLs from reproductively young (3 month) and old (9 months) ovaries. E) CellChat analysis of interaction between Gpnmb(+) macrophages and other immune SCLs. All analyses were derived from data reported by Isola and colleagues 2024 [18]. (TIF) [file pbio.3003204.s003.tif]

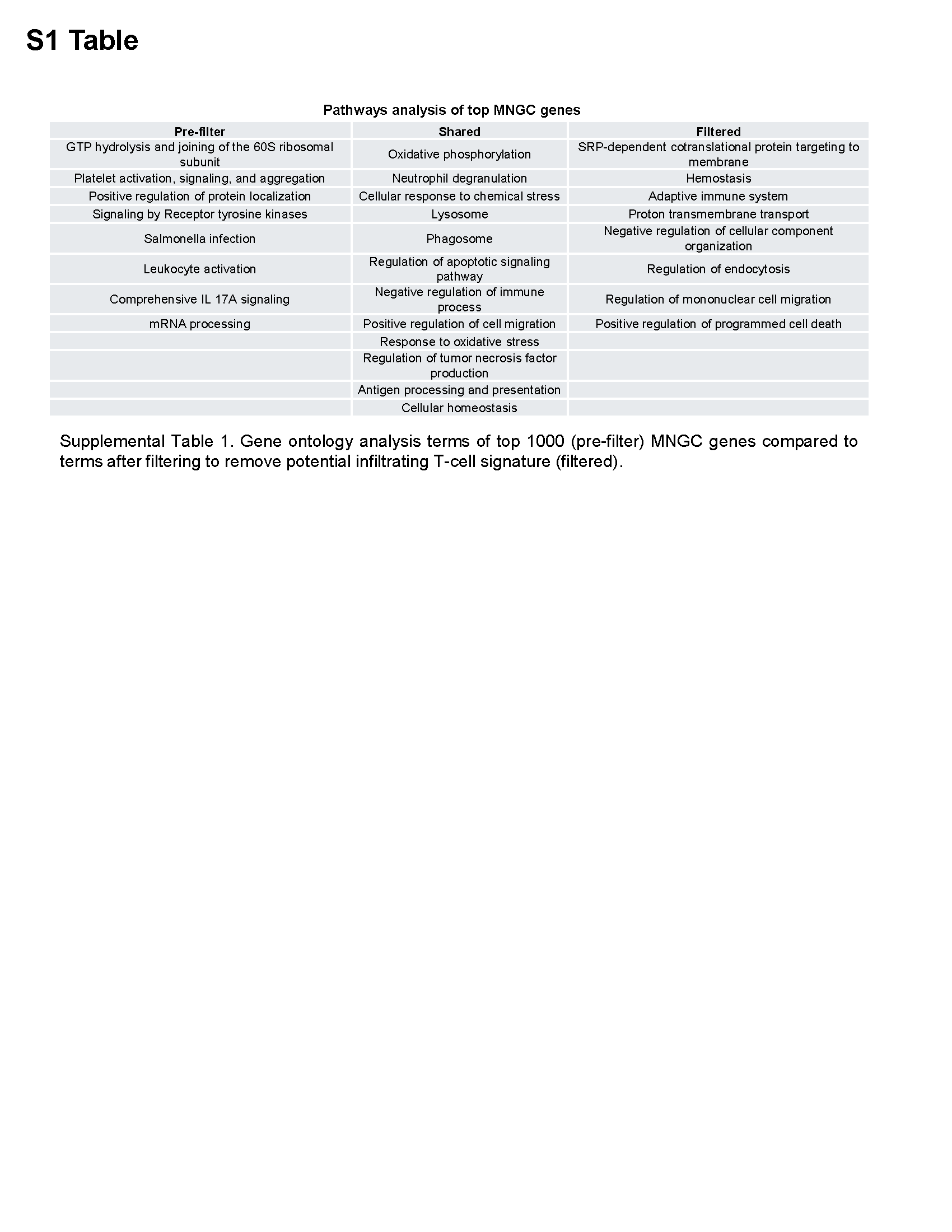

Supplement: S1 Table — (TIF) [file pbio.3003204.s004.tif]

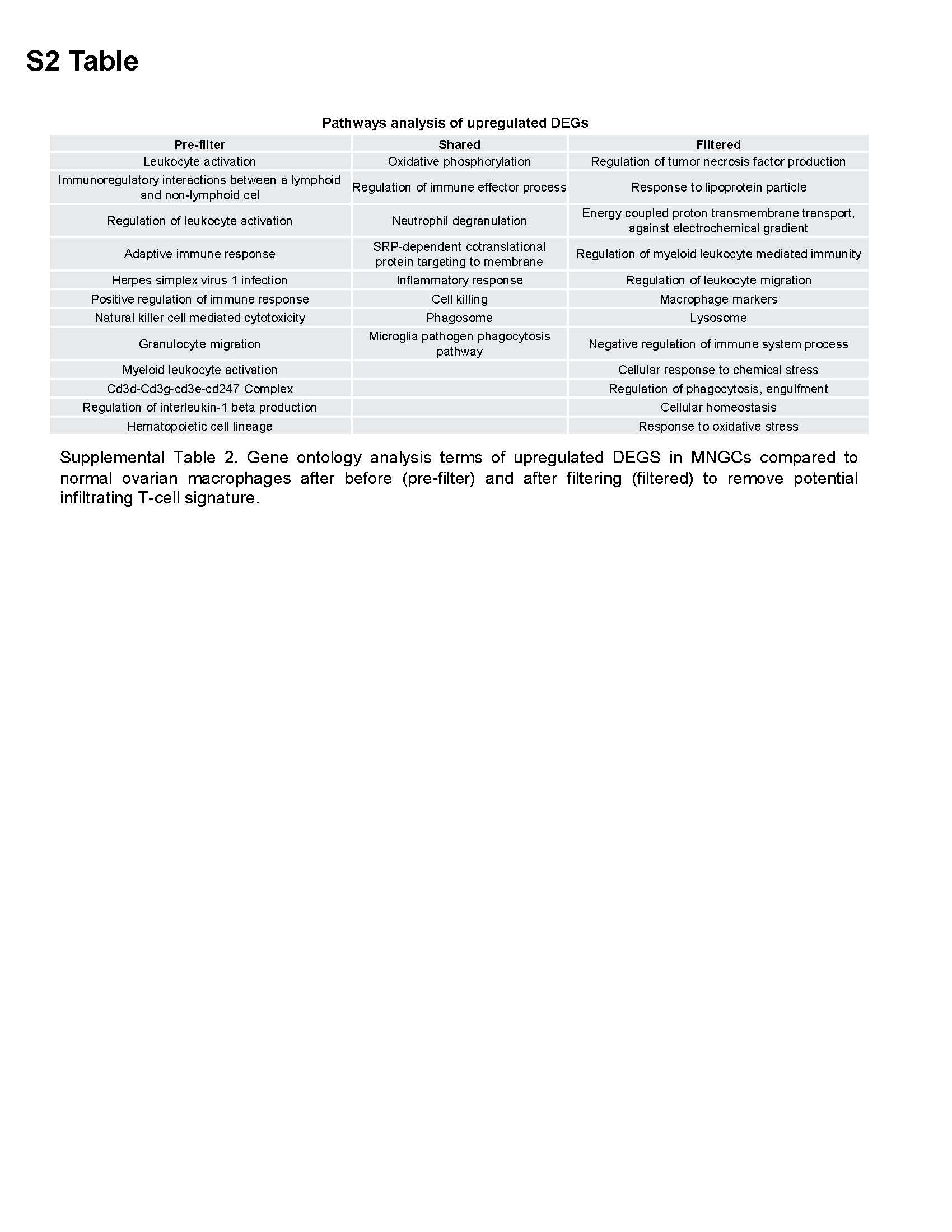

Supplement: S2 Table — (TIF) [file pbio.3003204.s005.tif]
