# Supplementary material for: Multinucleated giant cells are hallmarks of ovarian aging with unique immune and degradation-associated molecular signatures
Source: PLoS Biol. 2025 Jun 23;23(6):e3003204. doi: 10.1371/journal.pbio.3003204 (PMC12185119; doi:10.1371/journal.pbio.3003204)
Supplement: Attachment — Submitted filename: Response to reviewers.docx [file pbio.3003204.s014.docx]

We thank the reviewers for their valuable feedback which has served to strengthen our study. We are glad that they found our manuscript to be “novel” and “an interesting body of work.” Furthermore, it was acknowledged that we utilized technically advanced methods, our transcriptomic findings were well-validated, and that these results will provide key information for hypothesis generation and testing. We have addressed the suggestions comprehensively, and when relevant, modified the manuscript text accordingly. Changes to the manuscript text have been indicated as described below, and a version of the manuscript with tracked changes is included for ease of review. Line numbers have also been noted.

Rev. 1:

The authors have studied the presence and possible functions of multinucleated giant cells (MNGCs) in mouse and non-human primate ovaries. Their results show that MNGCs accumulate in the ovary with age. Using transcriptomic analysis of microdissected areas enriched for MNGCs, the authors validated that Gpnmb is one of the more highly expressed genes in MNGCs and that most expression of GPNMB is associated with MNGCs. Pathway analysis suggests potential functions of the MNGCs, notably when compared with the transcriptome of macrophages from young ovaries. MNGCs were found to co-localize with T cells and express CD4, suggesting possible interactions between these immune populations.

This is an interesting body of work. The findings are novel and have been generated by several technically advanced methods. Several observations from the transcriptomic analysis have been validated to strengthen the interpretation of the findings. The results suggest a number of possible functions and cell-cell interactions, which will require further testing to confirm.

Essential considerations:

1) The logic is not always clear, especially in the context of the potential significance of this work. For example, the Introduction indicates that MNGCs have degradative and proteolytic functions, and yet concludes that targeting this functionality might attenuate ovarian reproductive aging. It could be more easily argued that targeting these functions might actually accelerate reproductive aging.

Response: We agree that our analysis of the MNGC transcriptome points to unique roles in degradation which suggests that MNGCs may be compensating for the loss of other degradative processes or activity that occur with age. Thus, targeting MNGCs could exacerbate loss of ovarian tissue homeostasis and possibly accelerate ovarian aging. However, it is also possible that MNGCs may have negative consequences on the ovarian environment, especially given their unique immune signature and association with T-cells. Because the reasons for MNGC formation, as well as their effects on the ovarian environment and other cell types, currently remain unknown, the statement that MNGCs could be targeted to modulate ovarian aging is too speculative. Thus, we have revised the text to emphasize that further research is necessary to determine the drivers and consequences of ovarian MNGCs, as follows. “Thus, our study provides a robust framework for investigating MNGC biology across tissues, solidifies ovarian MNGCs as hallmarks of aging, and elucidates unique MNGC functionality which will set the stage for future studies understanding the impact of these cells in the ovary.” (lines 100-103) and “Understanding why MNGCs arise within the aging ovary and how they impact the ovarian microenvironment and other cell types will be crucial to determining whether these cells are a driver or a consequence of ovarian aging.” (lines 403-406)

2) Nowhere is it described how the MNGCs were quantified, as presented in Figure 1B and C. The Methods state only that "Trainable Weka Segmentation plugin in Fiji (NIH) was used to train the model on regions of non-ovary, non-MNGC ovary, and MNGCs". How were those regions identified or defined? How were clusters of macrophages distinguished from MNGCs? Histologically, the regions highlighted in mouse look very different than in the non-human primate.

Response: As requested, we have clarified and added further detail on how we identified MNGCs and quantified the area they occupied. MNGCs were identified both by their mutli-nucleation and by their characteristic brown pigmentation which is even visible in unstained sections as described: “MNGCs have an inherent brown pigment and are autofluorescent due to their lipofuscin content, which allows them to be visualized in ovarian tissue sections without specialized staining.” (lines 125-126). We have also provided additional details on the quantification methods as follows “To quantify the ovarian area occupied by MNGCs, the Trainable Weka Segmentation plugin in Fiji (NIH) was used to train the model on regions of the ovary with MNGCs identified by their multinucleation and brown pigmentation, regions of the ovary without MNGCs, and regions without ovarian tissue at all. The model was repeatedly trained until all areas were correctly segmented as validated by cross-referencing to an unsegmented hematoxylin-stained section. The percent MNGC area of an ovarian section was calculated as segmented MNGC area (red) out of total ovarian area (MNGC (red) + non-MNGC ovary segments (green)).” (lines 430-437).

Our method considers the area taken up by all pigmented cells, and thus we cannot exclude the possibility that other pigmented mono-nuclear cells/macrophages could have also been included. However, this is unlikely to be a major contribution since the majority of cells we quantified appeared mutli-nucleated. We have added the following clarifying text: “Although MNGCs contain lipofuscin which allows for their identification by brown pigmentation as well as autofluorescence, we cannot exclude the possibility that other lipofuscin-containing ovarian cells may be captured. Lipofuscin consists of oxidized proteins, lipids, and metals which increases with age and accumulates in senescent cells [19]. Thus, other ovarian cell types may also accumulate lipofuscin with age, and several studies have reported that lipofuscin-containing cells increase in the ovary with age [20,21]. These lipofuscin-containing cells correlate to regions of MNGCs, indicating the age-associated increase in lipofuscin is likely primarily due to MNGCs [18,20–22]. Further investigations are needed to determine whether other ovarian cell populations accumulate lipofuscin.” (lines 148-156).

As the reviewer noted, in the images we showed, the ovarian MNGCs in the non-human primate (NHP) differed morphologically from the mouse MNGCs. This is likely due to the decreased magnification of the NHP representative image compared to the mouse. We have since replaced the representative image of the NHP so that it is at the same scale as the mouse images. This comparison now better highlights the similarity of MNGC morphology between species.

3) Line 134: are autofluorescent (not autofluorescence). It also needs to be made clear if any other ovarian cell type is autofluorescent, as the authors use this feature to visualize MNGCs (Figure 1G and 1H).

Response: We corrected the text to indicate “autofluorescent.” The autofluorescence of MNGCs is due to their lipofuscin content. While we anticipate they are the primary lipofuscin-containing cells and autofluorescent structures within the ovary, we have included a discussion of the possibility of other non-MNGCs being detected by this imaging in technique in lines 148-156 as stated above.

4) Lines 205-207 rationalize the comparison of young ovarian macrophages vs. MNGCs "to minimize any confounding influence of age-associated changes". The argument could also be made that that choice focuses primarily on the effect of age, and not any differences between macrophages vs. MNGCs that are independent of age (i.e. if they had compared old macrophages vs. MNGCs). By comparing young macrophages with old MNGCs, the authors seem to be doing exactly what they state they are trying to avoid.

Response: The reviewer’s point is well taken. Ideally, we could have examined immuno-magnetic sorted ovarian macrophages from young and old animals for comparison to MNGCs. However, there was an added concern of using old animals that smaller MNGCs may make it through our cell filtering methods and, therefore, confound the signature of old “non-MNCG” macrophages. In support of this, the cells reported in Supplemental Fig. 3, which were captured in a single-cell RNA sequencing analysis of the mouse ovary (dataset derived by PMID: 38200272), are highly similar transcriptomically to MNGCs but made it through single-cell suspension filtering for analysis. This suggests that smaller MNGCs can pass through standard filtering procedures. Additionally, recent studies have compared the transcriptomes of ovarian macrophages from young and old animals and have not found profound transcriptomic differences in macrophages between the two age groups. We have further clarified our rationale in as follows, “Young ovarian macrophages were utilized as a control to ensure that no MNGCs were included in this cohort, as there is the potential of smaller MNGCs (<40 µm) from old ovaries to make it through single cell suspension filtering.” (lines 233-235).

We have also added a section describing prior work on ovarian macrophage characterization across reproductive age and how this relates to our findings as follows. “Ovarian macrophage populations have been recently defined utilizing RNA sequencing with single-cell and flow-cytometry based methods [4,8,9,18]. In most of these studies, macrophage number decreases or shows little change with age [4,8,9,18]. This decline in macrophage number may result from the transition and fusion of macrophages into MNGCs, which would result in an underestimate in the total ovarian macrophage population. Additionally, in these studies in which MNGCs are likely absent, comparison of young and old macrophages demonstrates minimal effect on their global transcriptome, irrespective of age. Therefore, MNGCs represent a distinct macrophage population that has not been effectively captured in existing studies to date.” (lines 296-304).

Minor comments:

5) Lines 219-220 conclude that these data confirm the macrophage identity of the F4/80 immuno-isolated cells and MNGCs, seemingly based on the common expression of a single pan-macrophage marker Adgre1. Is that correct?

Response: To confirm the macrophage identity of the immune-magnetic sorted macrophages and the LCM captured MNGCs, we provided expression profiles for a suite of genes that are expressed by all or subsets of macrophages. As *Adgre1* (the gene encoding the pan mouse macrophage marker, F4/80) is one of the most robust macrophage-specific markers, it was initially highlighted. Cd68 is a widely used macrophage marker as well and also showed significant enrichment in our macrophage populations compared to stroma, which we have now indicated: “Both macrophage populations had higher expression of the pan-macrophage marker *Adgre1* (F4/80) and the myeloid lineage marker *Cd68* compared to stroma samples.” (lines 243-245). We have also validated that the populations used for the transcriptomic analyses exhibit protein expression of F4/80 in the case of MNGCs (Fig. 1E) and phagocytic activity in the case of the immunoselected cells (Fig. 1D), further supporting their macrophage identity.

6) Line 290: "marker" not "maker".

Response: We have corrected this.

___________________________________________________________________________

Rev. 2:

This manuscript profiles and characterizes multinucleated giant cells (MNGC), which are found in aging mouse ovaries alongside chronic inflammatory signatures, but little is known about their function during reproductive aging. This group previously identified these cells in aged mouse ovaries. In this study, the authors characterize the presence of MNGC in aged mouse and non-human primate ovaries, using laser capture microdissection to generate a transcriptomic signature for these cells, and showing their interaction with Tcells. Overall, it is a straightforward manuscript that further provides expression data for these cells and highlights their potential role in cell degradation, energy production, and immune processes with aging. While descriptive, it does provide key information for hypothesis testing. I only have a few comments that I think would improve the manuscript:

Line 136. The statement about 'interconnected networks': Is it possible to visualize the 'networks' in a 3D rendering? The presence of MNGC throughout the ovary is obvious from the images, but less obvious is their 'interconnection'. This would add to the 'first 3D mapping' (line 140), which at the moment, is just a single video of a z-stack of autofluorescence in a mouse ovary - nice, but also, unclear variation between samples. Is there a 3D structure that they are following (vasculature? lymphatics?)

Response: We agree that the impact of the single 3D MNGC mapping was limited in our original manuscript. We have now included Z-stacks and corresponding 3D projections from three different mice (SV1, SV2, and SV3) as well as a 3D rendering of the original z-stack (SV4) and a large MNGC cluster (SV5). While we cannot perform a spatial analysis of MNGC proximity to specific features such as vasculature and lymphatics due to the lack of optimized protocols for overcoming MNGC autofluorescence to allow for co-staining and colocalization studies, this is an important future direction. However, our current imaging underscores that MNGCs are present throughout the three-dimensional space of the mouse ovary. We have modified the manuscript text as follows, since we cannot verify with our approach whether all MNGCs are interconnected: “Using this approach, we found that MNGCs formed clusters of various sizes throughout the ovary, with some clusters spanning hundreds of microns (Fig. 1G, SV1, SV2, SV3). These features are further exemplified through 3D rendering of the MNGC networks of a native ovary (SV4) and large MNGC cluster located in the white box in Fig. 1G (SV5). On a microscale, MNGCs were comprised of a complex autofluorescent cytoplasmic network containing numerous nuclei (white arrows) and were intercalated between non-autofluorescent tissue (Fig. 1H, SV6).” (Lines 140-146.)

Line 271. Of the MNGC 137 'unique genes', are these genes also expressed in MNGC in other contexts besides the ovary or are they unique to ovarian MNGC?

Response: This is a very interesting question. The list of unique MNGC genes is heavily influenced by T-cell populations that were captured upon laser capture microdissection, and thus may not be reflective of non-ovarian MNGCs. However, we compared the signature of ovarian MNGCs to other non-ovarian MNGCs. The most accessible non-ovarian MNGC models are those that are derived *in vitro*. Therefore, we compared our ovarian MNGC dataset to the top genes of a previously generated gene count list of *in vitro*-derived osteoclasts, foreign body giant cells, and Langhans giant cells. Ovarian MNGCs differ from these models, but do share some similarities such as high expression of *Gpnmb*. This analysis is included as a new Supplemental Fig. 1, and text describing these results has been added as follows “To determine how similar ovarian MNGCs are to models of non-ovarian MNGCs, we compared the top expressed ovarian MNGC genes to those of *in vitro* derived osteoclasts, foreign body giant cells, and Langhans giant cells utilizing a previously published transcriptomic dataset [24]. Of the top 100 ovarian MNGC genes that have orthologs between mice and humans, 39 are shared with at least one of the *in vitro*-derived MNGC models, whereas only 15 are conserved between all MNGC models (Supplemental Fig. 1A). The conserved top genes include various cathepsins, *Psap*, *Fth1*, *Vim*, *B2m*, *Grn*, *Gpnmb*, *Apoe*, *Cd63*, *Aplp2*, *Tmsb4x*, and *Hsp90ab1* (Supplemental Fig. 1B). Thus, while ovarian MNGCs shared some similarities with *in vitro*-derived MNGC models, the transcriptome of ovarian MNGC is largely distinct, indicating potential unique biology. Future studies to determine methods of *in vitro* ovarian MNGC derivation will be essential for further interrogating the biology of these unique MNGCs.” (lines 202-212).

Minor Comments

Figure 1, Panel H. What are the arrows? (add to legend).

Response: We have clarified what the arrows mean in the legend as follows: “Z-stack of autofluorescent MNGCs in cleared mouse ovaries at macro (G) and micro (H) scales, with white arrows in the last panel of (H) highlighting putative nuclei.” (lines 111-113). We have also clarified in lines 144-146: “On a microscale, MNGCs were comprised of a complex autofluorescent cytoplasmic network containing numerous nuclei (white arrows) and were intercalated between non-autofluorescent tissue (Fig. 1H, SV6).”

Line 283 and other places: use of the word, trends. "Trends" is a subjective measure with no statistical value and is used several times in the manuscript to report on presumably non-statistically significant data. "Trend" should generally be avoided in terms of hypothesis testing as it can be misleading. Instead, the p-value should be reported, and some statement on the study being underpowered, if this is the case.

Response: Thank you for this suggestion. We have included additional description of the statistics (within text and figure) or changed the language in instances of its use as follows. 1) In line 132, the p value and R^2^ are now indicated in the text “(R^2^=0.5; p=0.0513).” 2) We have changed the wording in lines 319-322 to “The other CD3 complex member *Cd3g* (p=0.08-0.11), the cytotoxic T-cell marker *Cd8a* (p=1.12-0.13), the helper T-cell marker *Cd4* (p=0.17-0.2), and other TCR constant chain genes (p=0.052-0.25) showed increased expression in MNGC samples compared to normal macrophage and stromal samples albeit not significant.” 3) We changed the wording in lines 358-359 to “MNGCs demonstrated a non-significant (p=0.10) increase in CD4 immunoreactivity compared to other ovarian compartments (Fig. 4H; Supplemental Fig. 2B).” 4) We have changed the wording in lines 367-370 to “*Gpnmb* was only expressed by a small fraction of the macrophage subcluster (Supplemental Fig. 3B-C) that were present in both young (3 m) and old (9 m) animals (Supplemental Fig. 3D), supporting that MNGC-like cells are represented in this fraction.“
